# Optimization of Naringin and Naringenin Extraction from *Citrus* × *paradisi* L. Using Hydrolysis and Excipients as Adsorbent

**DOI:** 10.3390/pharmaceutics14050890

**Published:** 2022-04-19

**Authors:** Jolita Stabrauskiene, Mindaugas Marksa, Liudas Ivanauskas, Jurga Bernatoniene

**Affiliations:** 1 Department of Drug Technology and Social Pharmacy, Lithuanian University of Health Sciences, LT-50161 Kaunas, Lithuania; jolita.stabrauskiene@lsmu.lt; 2 Institute of Pharmaceutical Technologies, Lithuanian University of Health Sciences, LT-50161 Kaunas, Lithuania; 3 Department of Analytical and Toxicological Chemistry, Lithuanian University of Health Sciences, LT-50161 Kaunas, Lithuania; mindaugas.marksa@lsmuni.lt (M.M.); liudas.ivanauskas@lsmuni.lt (L.I.)

**Keywords:** *Citrus* × *paradisi* L., grapefruit, flavanones, glycosides, aglycones, extractions, excipient, magnesium aluminometasilicate, adsorbent

## Abstract

While flavanones exist in a variety of chemical forms, their favorable health effects are most prominent in their free form—aglycones. Their concentrations in grapefruit (*Citrus* × *paradisi* L.) extracts vary according to the extraction and hydrolysis methods used. The primary aim of this work was to maximize the yields of naringin and naringenin from various parts of fresh grapefruit fruits (*flavedo*, *albedo*, *and segmental*) using different extraction and hydrolysis methods. In addition, we aimed to evaluate the excipient—magnesium aluminometasilicate—and determine its influence on the qualitative composition of grapefruit extracts. Extracts were obtained by heat reflux extraction (HRE), ultrasound-assisted extraction with an ultrasonic homogenizer (UAE*), and ultrasound-assisted extraction with a bath (UAE). Ultrasound-assisted extraction using a bath (UAE) was modulated using acidic, thermal, and alkaline hydrolysis. The highest yield of naringin 8A (17.45 ± 0.872 mg/g) was obtained from an *albedo* sample under optimal conditions using ultrasound-assisted extraction; a high yield of naringenin 23-SHR (35.80 ± 1.79 µg/g) was produced using the heat reflux method from the *segmental* part. Meanwhile, ultrasonic combined with thermal hydrolysis significantly increased flavanone extraction from the *albedo* and *segmental* parts: naringin from sample 9-A (from 17.45 ± 0.872 mg/g to 25.05 ± 1.25 mg/g) and naringenin from sample 15-S (from 0 to 4.21 ± 0.55 µg/g). Additionally, magnesium aluminometasilicate demonstrated significant increases of naringenin from all treated grapefruit parts. To our knowledge, this is the first report of magnesium aluminometasilicate used as an adsorbent in flavanone extractions.

## 1. Introduction

*Citrus* × *paradisi* L. is an essential member of the Citrus genus in the *Rutaceae* family. Grapefruit is a delicious fruit used in the juice and food industry. However, around half of all fruit waste is discarded as a waste product, even though it includes a huge number of biologically active components with distinct health benefits. Therefore, numerous studies have been conducted to extract and identify biologically active components present in various citrus fruits to understand the relationship between their presence in diet and health benefits and reduced risk of disease [1,2]. Citrus peels contain significant amounts of vitamin C, fiber, pectin, essential oils, and polyphenols. Therefore, they have high potential for use as value-adding products, particularly in the biotechnology and pharmaceutical industries [3,4].

Previous research has found that flavonoid types in citrus fruits varied between species and cultivars (Durand-Hulak et al., 2015), as did their contents and distribution in different fruit tissues (Antonio Cano et al., 2007) (p. 64, [5]) and [6].

Citrus peels are rich in phenolic components and essential flavonoids, which are widely studied and positively affect human health [7,8,9]. The main bioflavonoids in citrus fruits, flavanones narirutin, naringin, and aglycon naringenin, display high biological activity and antioxidant, anti-inflammatory, metabolic, antivirus, neuroprotective, and antitumor effects [10,11,12]. There is considerable evidence of how naringenin works synergistically with anticancer drugs, especially in resistant cancer forms. Therefore, the development of new pharmaceutical forms will significantly impact cancer treatment [11]. Other essential and beneficial flavonoids are hesperidin, rutin, diosmin, didymin, and quercetin.

**Figure 1:**
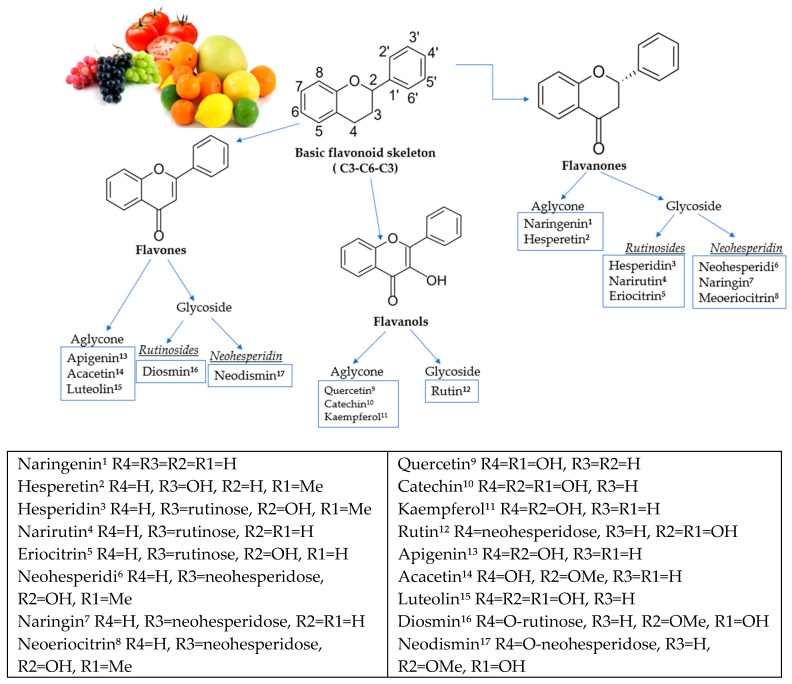
Molecular structures of flavonoids and their subclasses.

Flavanones in citrus fruits are in glycoside or aglycone forms [13]. Of the aglycone forms, the essential flavanones are naringenin and hesperetin. Glycosides are divided into two types: neohesperidosides (e.g., naringin), which have a bitter taste, and rutinosides (e.g., hesperidin, narirutin, and didymin) (Figure 1) [14,15]. The characteristic flavor of citrus fruits is caused by flavanones, usually diglycosides. The molecular structures of flavonoids are provided in Figure 1.

Natural flavanone glycosides, such as naringin, are not easily absorbed in intestinal absorptive cells because of their large hydrophilic structures—this reduces the expected effects of flavanones. As a result, the conjugated flavanones are inactive compounds but become active in aglycone form (naringenin). This bioflavonoid can be obtained from naringin hydrolysis with naringinase when the glucose molecule is removed from the structure [16].

**Figure 2:**
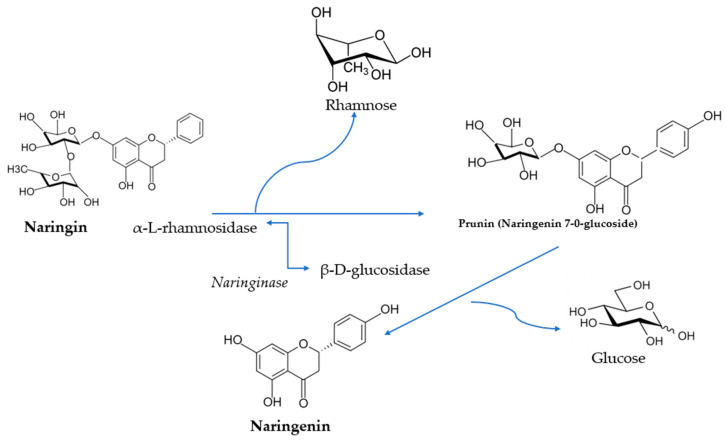
Hydrolysis of naringin by naringinase to produce naringenin.

Naringin is hydrolyzed into rhamnose and prunin by the naringinase exhibiting α-l-rhamnosidase activity, and then β-d-glucosidase catalyzes the hydrolysis of prunin to glucose and naringenin [17]. Hydrolysis of naringin to naringenin is shown in Figure 2.

Conventional extraction methods are based on the use of chemical solvents and the heating of the sample to maximize the solubility of active substances and accelerate their transfer. The extraction yield depends on various factors, including the type, concentration, and amount of the solvent used; its treatment time; and temperature. According to Sarah Luisa Rodriguez De Luna, 2020, the extraction time is a parameter that needs to be optimized in each experiment [18]. An increase in temperature may increase the release of flavonoids, but it also depends on the properties of the solvent used. The most appropriate extraction technique depends on the type of plant, so the defined selection criteria must be followed [19]. Different extraction methods, such as maceration, percolation, heat refluxing, Soxhlet extraction, supercritical extraction, microwave-assisted extraction, ultrasound-assisted extraction, and others, are the most commonly used methods for bioactive compound recovery from natural materials [20,21,22]. All these methods have their own advantages and disadvantages. However, maceration, percolation, continuous stirring, and Soxhlet extraction come with big disadvantages, such as long extraction times, complicated extraction operations, inflated costs, hazardous flammable liquid organic solvents, and large amounts of extraction solvents.

Meanwhile, green and sustainable extraction techniques, such as supercritical CO_2_ extraction method or ultrasound-assisted extraction, are environmentally friendly, safe, and non-toxic and are promising alternatives to conventional extraction methods [21,23,24]. 

Ultrasound-assisted extraction is widely used to extract biologically active compounds such as flavonoids, anthocyanins, and phenolic acids. According to Londono Londono et al. (2010), ultrasound-assisted extraction demonstrated a higher efficiency of flavonoid extraction from citrus peels in 60 min using methanol as a solvent than Soxhlet extraction with a more extensive solvent selection [22]. The biological activity of phenolic compounds is strongly dependent on the conditions of ultrasound-assisted extraction, such as temperature, type of solvent, and extraction time [21]. Cavitation is the primary action mechanism of ultrasound-assisted methods, causing cellular disruption, high solvent penetration, and particle size reduction.

Transformation of flavanone glycosides to aglycone can be achieved using an extraction method combined with hydrolysis. Chemical (using bases and acids) or thermal (high temperatures) hydrolysis can increase aglycon content [25,26].

The previous research demonstrated that magnesium aluminometasilicate can work as an adsorbent to increase the solubility of bioflavonoids in extracts [27,28].

Indeed, this study aimed to maximize the yields of naringin and naringenin from various parts of fresh grapefruit fruits using different extraction and hydrolysis methods, as well as to evaluate how magnesium aluminometasilicate affects the qualitative content of grapefruit extracts. 

## 2. Materials and Methods

### 2.1. Materials

**Figure 3:**
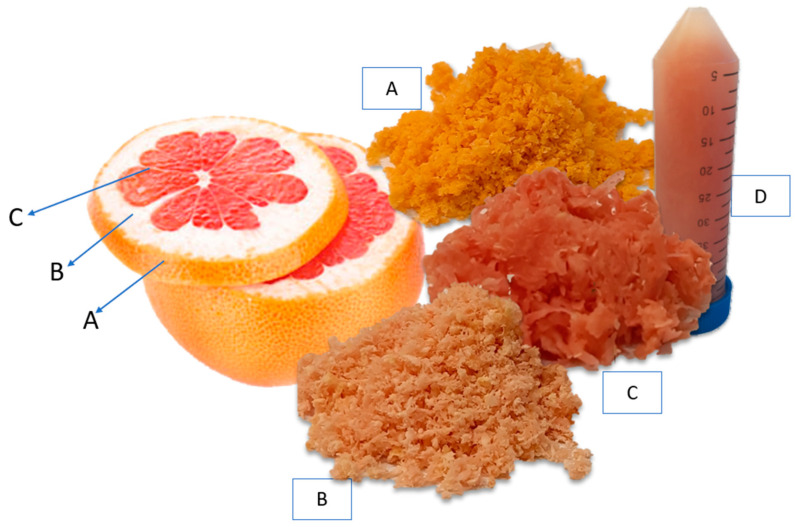
Fresh *Flavedo* (**A**), fresh *Albedo* (**B**), fresh *Segmental* (**C**), and frozen Juice (**D**) of *Citrus* × *paradisi* L.

The grapefruit fruits (*Citrus* × *paradisi* L., variety Star Ruby, Italy, region—unknown) were collected from the local market in Mastaičiai, Kaunas district, Lithuania. The fruit were separated into the *flavedo*, *albedo*, and *segmental* parts, then chopped up with a food processor and frozen in a freezer (−18 ± 0.9 °C) until extraction. The parts of the fruit used in this study are shown in Figure 3.

Magnesium aluminometasilicate (Neusilin^®^ US2, Fuji Chemical Industries Co., Ltd., Toyoma, Japan) was used as excipient. Ethanol 96% (Vilniaus degtine, Vilnius, Lithuania) was used as a solvent for extraction. 

### 2.2. Methods

Three different extraction techniques were used. In the first experiment, extracts were obtained by heat reflux extraction (Section 2.2.1), ultrasound-assisted extraction with an ultrasonic homogenizer (Section 2.2.3), and ultrasound-assisted extraction with a bath (Section 2.2.2). In the second experiment, the same methods and conditions were used with magnesium aluminometasilicate (Neusilin^®^), and in the third experiment, ultrasound-assisted extraction with a bath was modulated with acidic, thermal, and alkaline hydrolysis. The operational conditions for each extraction method are shown in Table 1. 

HPLC-grade and analytical-grade reagents were used: hydrochloric acid, sodium hydroxide, methanol, acetonitrile (Sigma Aldrich, Hamburg, Germany); standards of naringin and naringenin (Sigma Aldrich, Steinheim, Germany); and ethanol (96%) (Vilniaus Degtine, Vilnius, Lithuania).

#### 2.2.1. Heat Reflux Extraction (HRE)

The frozen, raw material was defrosted at room temperature and allowed to warm up to 25 ± 2 °C. A sample of 1 ± 0.05 g of defrosted grapefruit (*flavedo*, *albedo,* or *segmental* parts) was mixed with the solvent (70% ethanol (*v*/*v*)) at 1:10 ratio in a 250 mL round-bottom flask and refluxed in a sand bath at 100 ± 2 °C for one hour. The mixture was left to cool down at room temperature, and then centrifuged with Sigma 3-18K centrifuge (Sigma, Osterode am Harz, Germany) for 10 min at RCP 1789× *g*, followed by the decantation of the supernatant. The extracts were filtered through PVDF syringe filters (pore size 0.22 µm, Frisenette, Knebel, Denmark) prior to HPLC (high-performance liquid chromatography) analysis. All the extraction conditions are displayed in Table 1.

#### 2.2.2. Ultrasound-Assisted Extraction Bath (UAE)

Ultrasonic extraction was performed using an ultrasonic bath (Cambridge, UK, Grant Instruments ™ XUB12 Digital) (frequency of 38 kHz). A sample of 1 ± 0.05 g of defrosted *flavedo*, *albedo,* or *segmental* parts was macerated with the 50% or 70% ethanol solvent (*v*/*v*) at a ratio of 1:10, and extraction time (10 or 30 min), with the processing temperature of 50 ± 2 °C or 70 ± 2 °C (the temperature was regulated automatically by the ultrasonic bath). The mixture was allowed to cool down at room temperature (20 ± 5 °C) and then centrifuged with Sigma 3-18K centrifuge (Sigma, Osterode am Harz, Germany) for 10 min at RCP 1789× *g*, followed by the decantation of the supernatant. Next, the extracts were filtered through PVDF syringe filters (pore size 0.22 µm, Frisenette, Knebel, Denmark) before analyzing with HPLC (high-performance liquid chromatography). All the extraction conditions are displayed in Table 1.

#### 2.2.3. Ultrasound-Assisted Extraction Using an Ultrasonic Homogenizer (UAE*)

Ultrasound-assisted extraction was performed using an UP-250 ultrasonic homogenizer (frequency range 19–25 kHz, 250 W, probes amplitude 35 µm). Firstly, 5 ± 0.25 g of samples (*albedo*, *flavedo*, or *segmental* parts) were defrosted at room temperature before being mixed with the 70% ethanol solvent (*v*/*v*) at a 1:5 ratio in a 100 mL chemical beaker and extracted for 1, 3, and 5 min at a temperature of from 33.2 to 40 ± 2 °C. Next, the mixture was centrifuged with Sigma 3-18K centrifuge at room temperature (25 ± 5 °C) (Sigma, Osterode am Harz, Germany) for 10 min at RCF 3382× *g*, followed by the decantation of the supernatant. Then, the extracts were filtered through PVDF syringe filters (pore size 0.22 µm, Frisenette, Knebel, Denmark) before analyzing with HPLC (high-performance liquid chromatography). All the extraction conditions are displayed in Table 1.

#### 2.2.4. The Use of Magnesium Aluminometasilicate in the Preparation of Extracts 

Samples were modified with magnesium aluminometasilicate. The extracts were made under the same conditions as previously listed (Section 2.2.1 and Section 2.2.2). Again, 50% or 70% ethanol (*v*/*v*) or purification water was used as the solvent at a ratio of 1:10, and the excipient was added to the extraction mixture. The excipient concentration was 1% (*w*/*v*). Magnesium aluminometasilicate (g) was based on ethanol quantity. The samples were centrifuged for 10 min at RCF 1789× *g*, followed by decantation of the supernatant, and then the extracts were filtered through PVDF syringe filters (pore size 0.22 µm) before HPLC analysis. Sample preparation conditions are listed in Table 1.

### 2.3. Hydrolysis and Neutralization

#### 2.3.1. Acidic Hydrolysis and Neutralization Using *Albedo*, *Flavedo*, and *Segmental* Parts 

The applied modified acidic hydrolysis method was based on Keun Young Min et al.’s 2014 studies [29]. Firstly, the extracts were made under the previously listed conditions (Section 2.2.2). Then, 70% ethanol (*v*/*v*) was used as a solvent (ratio of 1:10), and the pH was adjusted with 2 M HCl to pH 2.5. After that, the extracts were sonicated using the UAE method at 50 ± 2 °C for 20 min. Next, the hydrolyzed extracts were allowed to cool down to 25 ± 2 °C and adjusted to pH 8 by adding an aqueous solution of 2 M NaOH while stirring. Finally, the neutralized extracts were centrifuged for 10 min at RCF 1789× *g* and filtered through PVDF syringe filters (pore size 0.22 µm) before HPLC analysis.

#### 2.3.2. Thermal Hydrolysis Using *Albedo*, *Flavedo*, and *Segmental* Parts

Firstly, we used an ultrasonic bath (frequency of 38 kHz) (Cambridge, UK, Grant Instruments ™ XUB12 Digital) to macerate 1 ± 0.05 g of *flavedo*, *albedo*, or *segmental* parts in 10 mL of 70% *v*/*v* ethanol solvent for a duration of 20 min at a temperature of 50 ± 2 °C (the temperature was regulated automatically by the ultrasonic bath). Thermal hydrolysis was completed by transferring the extract to a 250 mL round-bottom flask and refluxing in a sand bath at 100 ± 2 °C for 1 h. After cooling, the mixture was centrifuged for 10 min at 1789× *g* using a Sigma 3-18K centrifuge (Sigma, Osterode am Harz, Germany), followed by decantation of the supernatant. Before HPLC analysis, the extracts were filtered through PVDF syringe filters (pore size 0.22 m, Frisenette, Knebel, Denmark). The parameters of sample preparation are listed in Table 1.

#### 2.3.3. Alkaline Hydrolysis and Neutralization Using *Albedo*, *Flavedo*, and *Segmental* Parts

The applied modified alkaline hydrolysis method was based on Liuting Zhu’s 2020 studies. Firstly, 1 ± 0.05 g of raw material was macerated with 70% ethanol (*v*/*v*) (ratio of 1:10). Next, the pH was adjusted with 2 M NaOH to pH 10 (measured with Thermo Scientific Orion Versa Star™, an advanced electrochemistry meter). Next, the extracts were sonicated using the UAE method at 50 ± 2 °C for 20 min (Section 2.2.2). Finally, the neutralized extracts were centrifuged for 10 min at RCF 1789× *g*, followed by decantation of the supernatant. Before HPLC analysis, the extracts were filtered through PVDF syringe filters (pore size 0.22 m, Frisenette, Knebel, Denmark). The parameters of sample preparation are listed in Table 1.

### 2.4. Hydro Distillation (HD) 

Essential oil was extracted from the grapefruit peels (*flavedo* and *albedo* parts) using a hydro distillation technique. The procedure was as follows. Firstly, 44.5 ± 0.5 g of peels was placed in a round-bottom flask with 500 mL of distilled water and connected to a Clevenger’s distillation unit. Then, the essential oil was extracted by hydro distillation for 120 min. Next, the obtained essential oil, which was collected in a Clevenger’s receiver, was separated. Finally, the essential oil was stored in a glass bottle refrigerated at −4 °C until we determined the yield. Each extraction was performed three times under the same conditions. The yield of oil from the grapefruit peels’ *flavedo* and *albedo* parts (Y) obtained in each extraction was calculated by the formula:

Y (%) = Volume of essential oil (mL)/Amount of row material (g) × 100%(1)

### 2.5. HPLC–PDA Conditions

A Waters 2695 liquid chromatography with a photodiode array detector (Waters 996, 200–400 nm wavelength range) was used in the study. In addition, a chromatographic column ACE C18 (250 mm × 4.6 mm) with a sorbent particle size of 5 μm was used to separate the biologically active compounds.

**Figure 4:**
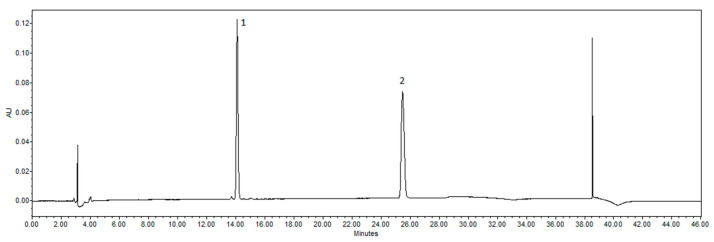
Chromatograms of standards detected by HPLC. Peaks identified: 1—naringin; 2—naringenin.

The following are the procedure details of HPLC method. The tested compounds were separated using gradient elution. Then, 10 µL of each extract was injected and analyzed at 280 nm. Eluent A: acetonitrile; eluent B: water at a rate of 1 mL/min. Gradient elution: 0.0 min, 10% A; 5 min, 20% A; 25 min, 40% A; 30 min, 100% A; 35 min, 100% A; 36 min, 10% A. The temperature of the column was 25 °C. The peaks were identified by comparing their UV-vis spectra and retention times to those of authentic reference standards. The samples were analyzed twice. The chromatograms of naringenin and naringin standards are shown in Figure 4.

The quantification and validation followed the methodical revision of natural products presented by Wolfender (2009) [30]. Standard stock solutions of primary concentrations of 100 µg/mL for naringin and naringenin were freshly prepared in 70% methanol, and calibration curves constructed using 6 different standard solution concentrations. Three injections per concentration were performed to determine linearity. Naringin and naringenin were plotted against the known concentrations of their associated standard solutions to establish calibration equations. A linear regression equation was calculated by the least-squares method. The regression coefficients of all calibration curves were R^2^ > 0.999, confirming the linearity of the concentration ranges.

The method sensitivity was evaluated by determining the limit of detection (LOD) and quantitation (LOQ). LOD and LOQ were calculated as the concentrations that gave signal-to-noise ratios of 3 to 10, respectively.

**Figure 5:**
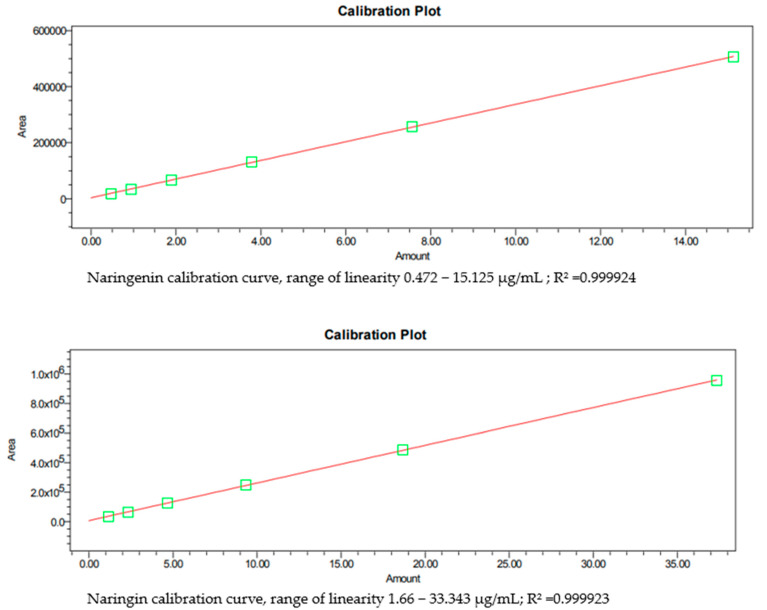
Naringin and naringenin calibration curves.

A standard mixture of naringin and naringenin was used for intra-day and inter-day precision testing. The method precision was demonstrated by performing five replicate non-consecutive injections of the usual mix on the same day on four different days. The results are reported in terms of RSD. In this study, standards (naringin and naringenin) were analyzed, and their retention time and spectra were compared with the prepared extracts. The linearity was determined by estimating the correlation coefficient R^2^ of the calibration curve (Figure 5) (naringin R^2^ = 0.99992, naringenin R^2^ = 0.99992), and the peak areas were used for quantification, Table 2. The linearity range of naringin was 1.166 to 33.343 µg/mL, and naringenin was 0.472 to 15.125 µg/mL. The results were expressed as µg/g and mg/g dry weight (DW) of naringenin and naringin, respectively.

### 2.6. Statistical Analysis

The data are presented as the mean and standard deviation (SD). Statistical analysis was performed with SPSS 20.0 (IBM Corporation, Armonk, NY, USA). One-way ANOVA was used to analyze the differences between extractions. In addition, post hoc comparisons of the means were conducted according to Tukey’s HSD test. The means of the compared samples were considered significantly different when *p* < 0.05. 

## 3. Results and Discussion

The extracts from fresh fruit materials were obtained using the UAE, HER, and UAE* methods (Table 1). Two different ethanol concentrations (50% and 70% *v*/*v*) were used for the extraction. Some of the samples were modified with magnesium aluminometasilicate.

The yield of naringin and naringenin was determined using HPLC-PDA.

### 3.1. Flavanone Determination in Citrus × paradisi L. Extracts

Naringin was obtained from all extracts of different parts (*flavedo, albedo*, and *segmental*) of fresh *Citrus* × *paradisi* L. fruit. The yields of naringin and naringenin obtained using different extraction methods are shown in Table 3.

Using the UAE extraction method, the highest yield of naringin was obtained from the *albedo* fraction using 50% ethanol (*v*/*v*) as a solvent with ultrasonic time of 30 min at 50 ± 2 °C, resulting in 8-A (17.45 ± 0.87 mg/g). In contrast, the lowest amount of naringin was obtained from the *segmental* part using 50% ethanol (*v*/*v*) with ultrasonic time of 30 min at 50 ± 2°C—17-S (4.31 ± 0.96 mg/g) (*p* < 0.05). Meanwhile, naringenin was found only in *albedo*, and its highest concentration was detected using 70% ethanol (*v*/*v*) for 30 min at 50 ± 2 °C, 12-A (4.63 ± 0.23 µg/g).

When the effect of ethanol concentration (50% or 70% (*v*/*v*)) was analyzed, it was discovered that 70% (*v*/*v*) ethanol produced better results in some cases. For example, samples taken from *flavedo* parts had increased naringin from 7-A (14.79 ± 0.73 mg/g) to 9-A (17.39 ± 1.10 mg/g), and the *albedo* part had increased naringenin from 7-A (3.36 ± 0.16 µg/g) to 9-A (4.57 ± 0.22 µg/g) (50% and 70% (*v*/*v*) ethanol, respectively) (*p* < 0.05).

The differences between samples 7-A and 8-A (shown in Table 3 with 50% (*v*/*v*) ethanol showed that an increase in the sonication time statistically significantly increased flavanone yield from the *flavedo* part, 14.79 ± 0.73 mg/g to 17.45 ± 0.87 mg/g and 3.36 ± 0.168 µg/g to 3.55 ± 0.17 µg/g (naringin and naringenin, respectively). 

When compared to the UAE method, the HRE extraction method produced a significant yield, with naringenin qualitatively doubled in extracts from the *albedo* 22-AHR (12.60 ± 0.63 µg/g) and *segmental* parts 23-SHR (35.80 ± 1.77 µg/g).

In the case of the UAE* method, the maximum yield was obtained when naringenin was released from the segmental part using 70% (*v*/*v*) ethanol in a temperature range of 33.5 to 40 ± 2 °C; the highest amount of naringenin detected was from the sample 28-SUX2 (7.40 ± 0.37 µg/g).

Unfortunately, the supercritical CO_2_ extraction method from fresh fruit flavedo did not produce statistically meaningful results (total amount of extract 0.79 ± 0.039 g/100 g) (*p* > 0.05). However, given that the findings were not statistically significant, they are not included in Table 3.

#### 3.1.1. Flavanones Extraction Using the UAE Method with Acidic, Alkaline, and Thermal Hydrolysis

**Figure 6:**
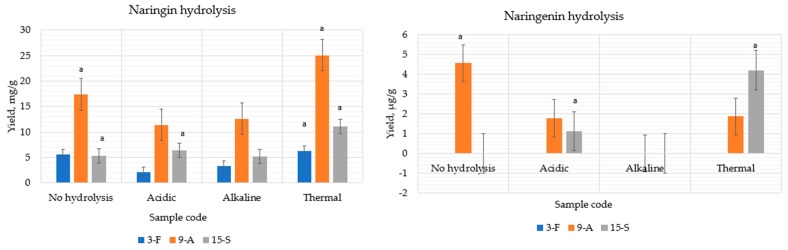
Comparison of the extraction yields of naringin and naringenin obtained with and without hydrolysis. ᵃ *p* < 0.05 when compared to extraction without hydrolysis. Extract ID and preparation conditions are displayed in Table 1.

The extracted sample was prepared using the UAE method with acidic, alkaline, and thermal hydrolysis and 70% (*v*/*v*) ethanol as a solvent (Section 2.3.1, Section 2.3.2 and Section 2.3.3). Using UAE with thermal hydrolysis, naringin yields were increased from all parts, for example, from the *flavedo* part (from 5.59 ± 0.279 mg/g to 6.25 ± 0.312 mg/g), doubled yield from the *albedo* part (from 17.39 ± 0.869 mg/g to 25.05 ±1.25 mg/g), and from the *segmental* part (from 5.26 ± 0.263 mg/g to 11.07 ± 0.55 mg/g) (Table 4). The highest amount of aglycon naringenin was obtained from the *segmental* part using thermal and acidic hydrolysis: 0–1.12 ± 0.056 µg/g–4.21 ± 0.21 µg/g (UAE without hydrolysis, acidic, and thermal hydrolysis, respectively) (*p* < 0.05 when compared to extraction without hydrolysis). Statistically significant results are shown in Figure 6.

Wenbin Chen et al. (2017) demonstrated that the complete hydrolysis of the flavonoid glycosides was achieved only by refluxing at 80 ± 2 °C [31]. This tendency was also observed in this study using thermal hydrolysis.

#### 3.1.2. Flavanone Extraction Using an Excipient as Adsorbent 1% Magnesium Aluminometasilicate

Using excipients as adsorbent may improve the solubility of certain active substances in drugs that have poor water solubility [27,32]. Therefore, in this study, we decided to use excipients during extractions to determine whether they could increase the yields of flavanones. The used heat reflux extraction and ultrasound-assisted extraction bath methods obtained most flavanones (Section 2.2.4). These extraction conditions using additional compounds were applied to improve the solubility of flavanones. 

**Figure 7:**
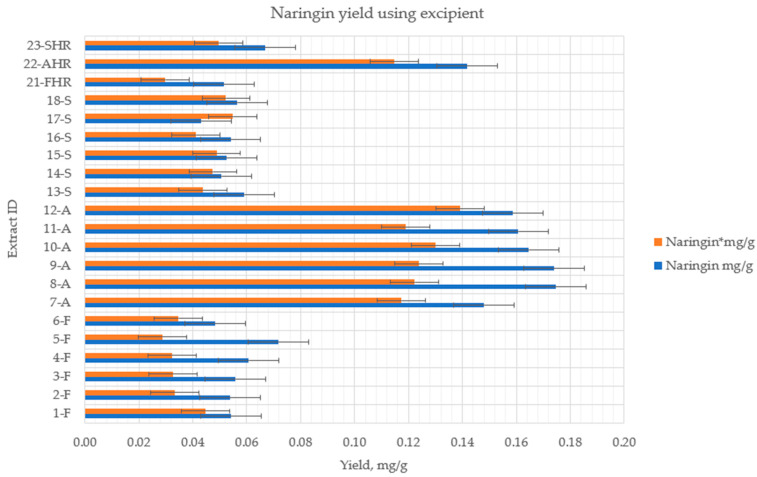
The quantitative yield of flavanone glycosides using excipient (1%). *p* < 0.05 when control samples without excipients were compared to samples with magnesium aluminometasilicate *. Extract ID and preparation conditions are displayed in Table 1.

**Figure 8:**
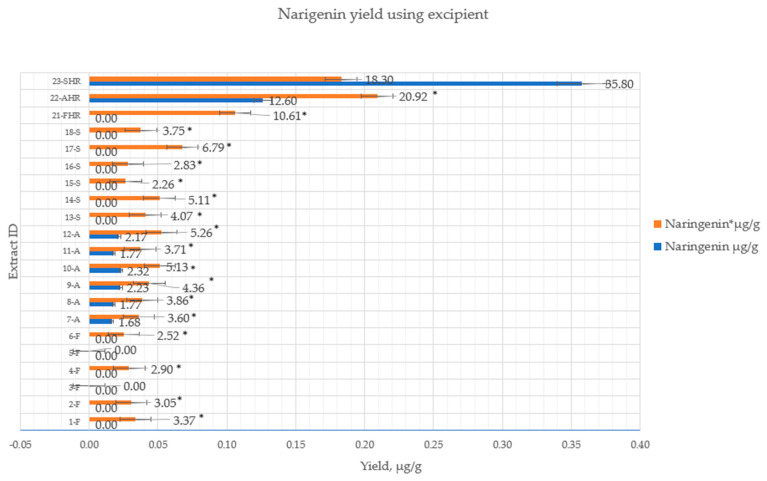
The quantitative yield of flavanone aglycones using excipients (1%). *p* < 0.05 when control samples without excipients were compared to samples with magnesium aluminometasilicate *. Extract ID and preparation conditions are displayed in Table 1.

The use of magnesium aluminometasilicate during extraction significantly affected the release of naringenin in all samples. There was a statistically significant improvement in naringenin self-efficacy in the HRE from the *flavedo* (samples 21-FHR—2.92 ± 0.503 µg/g). There was also a statistically significant increase of naringenin yield in samples from *segmental* parts using the UAE method: 13-S (4.07 ± 0.203 µg/g); 14-S (5.11 ± 0.257 µg/g); 15-S (2.66 ± 0.133 µg/g); 16-S (2.83 ± 0.141 µg/g); 17-S (6.78 ± 0.34 µg/g); 18-S (3.75 ± 0.19 µg/g). The operational conditions for each extraction method are shown in Table 1. The quantitative yield of flavanone glycosides and aglycone using magnesium aluminometasilicate are shown in Figure 7 and Figure 8.

Using the UAE technique (50 ± 2 °C, 30 min sonication time, and the solvent of purified water with 1% magnesium aluminometasilicate), naringenin was detected in samples from the *flavedo* part, with a yield of 2.38 ± 0.119 µg/g. However, given that the findings were not statistically significant, they are not included in Table 3.

## 4. Conclusions

The results indicated that the highest yields of naringin can be obtained using an ultrasound-assisted extraction bath with optimal conditions (50% of ethanol *v*/*v* as a solvent, sonication time 30 min at 50 ± 2 C°) from the *albedo* part 8-A (17.45 ± 0.872 mg/g); meanwhile, the highest yield of naringenin was obtained by heat reflux extraction method from the *segmental* part with 70% of ethanol (*v*/*v*) 23-SHR (35.80 ± 1.79 µg/g). Significant results of naringenin yield in terms of ultrasound-assisted extraction using an ultrasonic homogenizer were obtained using extracts from the *segmental* part—in a short time (3 min), the quantity of naringenin increased up to 28-SUX2 (7.40 ± 0.37 µg/g) compared with UAE method (Table 3).

The solvent, extraction time, and temperature influenced the recovery of flavanones from fruit materials. The high temperatures could increase active compound quantities, so using the UAE method combined with thermal hydrolysis, the amount of naringin in the *segmental* and *albedo* portions was at least doubled: 15-S (11.07 ± 0.55 mg/g); 9-A (25.05 ± 1.25 mg/g). The acidic and thermal hydrolysis influenced the amount of aglycon naringenin from the *segmental* part and demonstrated better quantification when compared with extraction without hydrolysis, (0 µg/g–1.12 ± 0.056 µg/g–4.21 ± 0.21 µg/g) (Table 4).

The magnesium aluminometasilicate, which was used as an adsorbent to increase flavanone yields from different fruits parts, increased the amount of naringenin in all samples from the *flavedo*, *albedo,* and *segmental* parts, using 50% or 70% of ethanol (*v*/*v*). Meanwhile, when purified water was used as the solvent, naringenin was detected in a small amount from the *flavedo* part (2.38 ± 0.119 µg/g). Thus, we could achieve the same quantity of aglycone with a lower concentration of solvent (ethanol) when using additional adsorbent magnesium aluminometasilicate. However, the quantity of naringin was reduced by 15% in extracts from different parts of the fruits (Figure 7).

After reviewing the studies, we found that flavanone solubility may be increased in the aqueous solvent or a lower concentration of ethanol by adding additional agents, such as cyclodextrin, to improve the release and stability of the active compounds.

The majority of the active compounds were extracted from the *Citrus* × *paradisi* L. *albedo* and *segmental* parts using the UAE and HRE methods, so these parts are a promising material for further research and can be used to develop novel pharmaceutical products.

## Figures and Tables

**Table 1:** Operational conditions of the different extraction methods.

Extract ID	Extraction Temp. °C	Extraction Time, min	Material:Solvent Ratio (g/mL)	Solvent	Excipient	Hydrolysis Methods
	***HRE—Heat-Reflux Extraction***					
21-FHR				Ethanol 70% (*v*/*v*)	*Magnesium* *aluminometasilicate*	-
22-AHR	100 ± 2	60	1:10			-
23-SHR						-
	***UAE—Ultrasound-Assisted Extraction Bath***					
1-F	50 ± 2	20	1:10	Ethanol 50% (*v*/*v*)	*Magnesium* *aluminometasilicate*	-
2-F	50 ± 2	30	1:10	Ethanol 50% (*v*/*v*)		-
3-F	50 ± 2	20	1:10	Ethanol 70% (*v*/*v*)		AC*/AL*/T*
4-F	50 ± 2	30	1:10	Ethanol 70% (*v*/*v*)		-
5-F	70 ± 2	30	1:10	Ethanol 50% (*v*/*v*)		-
6-F	70 ± 2	30	1:10	Ethanol 70% (*v*/*v*)		-
7-A	50 ± 2	20	1:10	Ethanol 50% (*v*/*v*)		-
8-A	50 ± 2	30	1:10	Ethanol 50% (*v*/*v*)		-
9-A	50 ± 2	20	1:10	Ethanol 70% (*v*/*v*)		AC*/AL*/T*
10-A	50 ± 2	30	1:10	Ethanol 70% (*v*/*v*)		-
11-A	70 ± 2	30	1:10	Ethanol 50% (*v*/*v*)		-
12-A	70 ± 2	30	1:10	Ethanol 70% (*v*/*v*)		-
13-S	50 ± 2	20	1:10	Ethanol 50% (*v*/*v*)		-
14-S	50 ± 2	30	1:10	Ethanol 50% (*v*/*v*)		-
15-S	50 ± 2	20	1:10	Ethanol 70% (*v*/*v*)		AC*/AL*/T*
16-S	50 ± 2	30	1:10	Ethanol 70% (*v*/*v*)		-
17-S	70 ± 2	30	1:10	Ethanol 50% (*v*/*v*)		-
18-S	70 ± 2	20	1:10	Ethanol 70% (*v*/*v*)		-
	UAE*—Ultrasound-Assisted Extraction Using an Ultrasonic Homogenizer					
27-SUX1	from 33.2 to 40 ± 2	1	1:5	Ethanol 70% (*v*/*v*)	-	-
28-SUX2	from 33.2 to 40 ± 2	3	1:5	Ethanol 70% (*v*/*v*)	-	-
29-SUX3	from 33.2 to 40 ± 2	5	1:5	Ethanol 70% (*v*/*v*)	-	-
30-FUX1	from 33.2 to 40 ± 2	1	1:5	Ethanol 70% (*v*/*v*)	-	-
31-FUX2	from 33.2 to 40 ± 2	3	1:5	Ethanol 70% (*v*/*v*)	-	-
32-FUX3	from 33.2 to 40 ± 2	5	1:5	Ethanol 70% (*v*/*v*)	-	-
33-AUX1	from 33.2 to 40 ± 2	1	1:5	Ethanol 70% (*v*/*v*)	-	-
34-AUX2	from 33.2 to 40 ± 2	3	1:5	Ethanol 70% (*v*/*v*)	-	-
35-AUX3	from 33.2 to 40 ± 2	5	1:5	Ethanol 70% (*v*/*v*)	-	-

UAE—ultrasound-assisted extraction bath, UAE*—ultrasound-assisted extraction using an ultrasonic homogenizer; HRE—heat reflux extraction. AC*—acidic hydrolysis, AL*—alkaline hydrolysis, T*—thermal hydrolysis.

**Table 2:** The linearities of calibration curves of flavanones.

Component	Calibration Equation	Coefficient of Determination *R*^2^	Coefficient of Correlation *R*	LOD* µg/mL	LOQ** µg/mL
Naringin	Y = 25.500x + 6720	0.999923	0.99996	0.146	0.583
Naringenin	±Y = 33.300x + 3570	0.999924	0.99996	0.118	0.430

LOD*—limit of detection; LOQ**—limit of quantification.

**Table 3:** Yields of flavanones recovered using different extraction methods.

Extraction Methods	Extract ID *	Naringin mg/g	Naringenin µg/g
*Ultrasound-assisted extraction bath*	1-F	5.41 ± 0.27 ᵈ	-
	2-F	5.38 ± 0.267	-
	3-F	5.59 ± 0.279 ᵈ	-
	4-F	6.08 ± 0.304	-
	5-F	7.18 ± 0.359	-
	6-F	4.82 ± 0.241 ᵇ	-
	7-A	14.79 ± 0.739 ᵈ	3.36 ± 0.168 ᵈ^,^ᵇ
	8-A	17.45 ± 0.872	3.55 ± 0.1775 ᵇ
	9-A	17.39 ± 0.869 ᵈ	4.57 ± 0.228 ᵈ^,^ᵇ
	10-A	16.46 ± 0.823	4.63 ± 0.231 ᵇ
	11-A	16.08 ± 0.820	3.53 ± 0.176 ᵇ
	12-A	15.86 ± 0.793	4.34 ± 0.207 ᵇ
	13-S	5.91 ± 0.295 ᵇ^,^ᵈ^,^ᵉ	- ᵇ^,^ᵉ
	14-S	5.06 ± 0.253 ᵇ^,^ᵉ	- ᵇ^,^ᵉ
	15-S	5.26 ± 0.263 ᵈ^,^ᵇ^,^ᵉ	- ᵇ^,^ᵉ
	16-S	5.40 ± 0.27 ᵇ^,^ᵉ	- ᵇ^,^ᵉ
	17-S	4.31 ± 0.215 ᵇ^,^ᵉ	- ᵇ^,^ᵉ
	18-S	5.65 ± 0.282 ᵇ^,^ᵉ	- ᵇ^,^ᵉ
*Heat reflux* *extraction*	21-FHR	5.16 ± 0.258 ᵃ	-
	22-AHR	14.17 ± 0.708 ᵃ	12.60 ± 0.63
	23-SHR	6.68 ± 0.334	35.80 ± 1.79
*Ultrasound-assisted extraction using an ultrasonic homogenizer*	27-SUX1	5.15 ± 0.257 ᵃ^,^ᵇ	4.39 ± 0.219
	28-SUX2	6.38 ± 0.319 ᵇ	7.40 ± 0.37
	29-SUX3	5.56 ± 0.279 ᵃ^,^ᵇ	5.88 ± 0.294
	30-FUX1	0.96 ± 0.048 ᵃ^,^ᵇ	-
	31-FUX2	1.05 ± 0.0525 ᵃ^,^ᵇ	-
	32-FUX3	0.98 ± 0.049 ᵃ^,^ᵇ	-
	33-AUX1	5.75 ± 0.287 ᵃ^,^ᵇ	- ᵃ^,^ᵇ
	34-AUX2	6.67 ± 0.333 ᵃ^,^ᵇ	- ᵃ^,^ᵇ
	35-AUX3	6.13 ± 0.306 ᵃ^,^ᵇ	- ᵃ^,^ᵇ

* The meanings of the abbreviations are presented in Table 1. ᵈ *p* < 0.05 when UAE with 50% ethanol (*v*/*v*) were compared with UAE with 70% ethanol (*v*/*v*). ᵃ *p* < 0.05 vs. ultrasound-assisted extraction bath; ᵇ *p* < 0.05 vs. heat reflux extraction; ᵉ *p* < 0.05 vs. ultrasound-assisted extraction using an ultrasonic homogenizer.

**Table 4:** Yields of flavanones recovered using UAE extraction methods with and without hydrolysis.

	*Naringin* mg/g				*Naringenin* µg/g			
Extract ID **	No Hydrolysis	AC *	AK *	T *	No Hydrolysis	AC *	AK *	T *
3-F	5.59 ± 0.279 ᵃ	2.14 ± 0.10	3.36 ± 0.168	6.25 ± 0.312 ᵃ	-	-	-	-
9-A	17.39 ± 0.869 ᵃ	11.39 ± 0.56	12.59 ± 0.629	25.05 ± 1.25 ᵃ	4.57 ± 0.249	1.78 ± 0.089	-	1.87 ± 0.09
15-S	5.26 ± 0.263 ᵃ	6.39 ± 0.319	5.13 ± 0.256	11.07 ± 0.55 ᵃ	0 ᵃ	1.12 ± 0.065	-	4.21 ± 0.21 ᵃ

* The meanings of the abbreviations are in Table 1. ** The meanings of the abbreviations are in Table 1. ᵃ *p* < 0.05 when compared to extraction without hydrolysis.

## Data Availability

Not applicable.
